# Catheter ablation versus medical therapy for ventricular tachycardia in patients with ischemic heart disease: A systematic review and meta-analysis of randomized controlled trials

**DOI:** 10.1016/j.ipej.2025.03.004

**Published:** 2025-03-07

**Authors:** Ubaid Khan, Yehya Khlidj, Ahmed A. Ibrahim, Ahmed Mazen Amin, Mohamed Saad Rakab, Majd M. AlBarakat, Muhammad Haris Khan, Zuhair Majeed, Muhammad Imran, Junaid Ali, Chet RanaBhat, Wajeeh Ur Rehman, Justin Brilliant, Kashif Chaudhry

**Affiliations:** a Division of Cardiology, University of Maryland School of Medicine, Baltimore, MD, USA; b Faculty of Medicine, Algiers University, Alger Centre, Algeria; c Faculty of Medicine, Menoufia University, Menoufia, Egypt; d Faculty of Medicine, Mansoura University, Mansoura, Egypt; e Faculty of Medicine, Jordan University of Science and Technology, Irbid, Jordan; f Department of Medicine, Saidu Medical College, Swat, Pakistan; g Department of Medicine, King Edward Medical University, Lahore, Pakistan; h Faculty of Medicine, University College of Medicine and Dentistry, The University of Lahore, Lahore, Pakistan; i Department of Medicine, Saint Peter's University Hospital, New Brunswick, NJ, USA; j Department of Medicine, University of Maryland Medical Center Midtown Campus, Baltimore, MD, USA; k Department of Internal Medicine, United Health Services, NY, USA; l Department of Electrophysiology, UPMC Williamsport, Pennsylvania, USA

**Keywords:** Ventricular tachycardia, Ischemic heart disease, Catheter ablation, Intracardiac cardioverter defibrillator, Meta-analysis

## Abstract

**Background:**

Ventricular tachycardia (VT) is a common chronic complication of ischemic heart disease (IHD), even in the era of contemporary coronary intervention. The use of implantable cardioverter-defibrillators (ICDs) has reduced mortality, but ICD shocks can be painful and traumatizing. Catheter ablation has been posited to reduce VT incidence and is commonly used in IHD patients when antiarrhythmic drugs do not suppress VT.

**Purpose:**

We aim to review the clinical efficacy and safety of catheter ablation vs medical therapy in patients with IHD.

**Methods:**

We conducted comprehensive searches across PubMed, CENTRAL, Web of Science, Scopus, and Embase until May 2024. Pooled data were reported using risk ratio (RR) for dichotomous outcomes and mean difference (MD) for continuous outcomes. This systematic review and meta-analysis was registered with PROSPERO ID: CRD42024551760.

**Results:**

We included eight RCTs with a total of 1252 patients. Patients who underwent catheter ablation had a lower risk of VT storms compared to those who received medical therapy alone [RR: 0.74 with 95 % CI: (0.60, 0.91), P = 0.005), Compared to medical therapy, the catheter ablation group also required less appropriate ICD therapy [RR: 0.72 with 95 % CI: (0.57, 0.90), P = 0.005), and fewer appropriate ICD shocks [RR: 0.75 with 95 % CI: (0.57, 0.99), P = 0.04). However, there was no significant difference in Ventricular tachycardia/Ventricular fibrillation (VT/VF) recurrence [RR: 0.94 with 95 % CI: (0.83, 1.06), P = 0.33) and all-cause mortality [RR: 0.87 with 95 % CI: (0.70, 1.09), P = 0.22).

**Conclusion:**

Catheter ablation is associated with a significant reduction in ventricular storms, appropriate ICD therapy, and appropriate ICD shocks while demonstrating similar safety in managing VT in IHD patients compared to medical therapy alone.

## 1 Introduction

Implantable cardioverter-defibrillators (ICDs) are a cornerstone in the prevention of sudden cardiac death in patients with ventricular arrhythmias. However, ICD shocks, although lifesaving, can significantly impact the patient's quality of life, often resulting in anxiety, depression, and even post-traumatic stress disorder [1,2]. Reducing the burden of ICD therapies is a critical goal in managing patients with these devices. Two primary strategies to achieve this are antiarrhythmic drug (AAD) therapy and catheter ablation. This meta-analysis aims to evaluate the effectiveness of ablation versus AADs in reducing the ICD therapy burden.

The need for this analysis stems from the mixed results observed in previous studies regarding the efficacy of AADs and catheter ablation. AADs, while effective in suppressing arrhythmias, often come with significant side effects and long-term tolerability issues [3,4]. Drugs like amiodarone, sotalol, and mexiletine are commonly used but are not without risks, including proarrhythmic effects [5]. Moreover, the psychological burden of anticipating shocks remains, as AADs do not entirely eliminate the occurrence of arrhythmias.

On the other hand, catheter ablation has emerged as a promising alternative by directly targeting and modifying arrhythmogenic substrates within the heart. Studies such as the SMASH-VT trial have demonstrated that substrate-based ablation can significantly reduce the incidence of ICD therapies compared to standard medical therapy alone [6]. The premise of catheter ablation is to eliminate the electrical circuits causing arrhythmias, thereby reducing the frequency of ICD interventions and potentially improving the patient's quality of life and clinical outcomes [7].

Previous trials have shown variable outcomes regarding the superiority of one approach over the other. The VANISH trial, for instance, highlighted the benefits of catheter ablation in patients with recurrent ventricular tachycardia (VT) despite AAD therapy, showing a significant reduction in arrhythmia recurrence and ICD shocks [8]. However, the study also underscored the procedural risks associated with ablation, such as cardiac tamponade and vascular complications [9].

This meta-analysis consolidates evidence from multiple trials to provide a more definitive comparison of ablation versus AAD therapy. By examining a larger pooled population, this study aims to address the limitations of individual studies, such as small sample sizes and heterogeneity in patient populations and study designs. This comprehensive analysis will include data from pivotal trials like CALYPSO, SURVIVE-VT, VTACH, SMS, and others, each contributing valuable insights into the efficacy and safety of both treatment strategies.

Furthermore, the meta-analysis will explore secondary outcomes such as overall mortality, heart failure hospitalizations, and quality of life metrics [10]. These outcomes are crucial as they provide a broader perspective on the impact of each treatment modality beyond the primary endpoint of reducing ICD therapies.

This meta-analysis aims to inform clinical decision-making by providing robust evidence on the optimal strategy for reducing the ICD burden in patients with ventricular arrhythmias. The findings will have significant implications for guiding treatment choices, improving patient outcomes, and potentially influencing guidelines and standard care practices in managing ventricular arrhythmias in ICD recipients.

## 2 Methodology

### 2.1 Protocol registration

The present systematic review and meta-analysis followed the guidelines outlined in the Preferred Reporting Items for Systematic Reviews and Meta-Analyses (PRISMA) [11] and the Cochrane Handbook of Systematic Reviews and Meta-Analysis [12]. Our review procedure was registered and published in PROSPERO with ID: CRD42024551760.

### 2.2 Data sources & search strategy

Two reviewers (U.K. & M.I.) conducted a systematic search across the Web of Science, Scopus, PubMed , Embase, and Cochrane Central Register of Controlled Trials (CENTRAL) from inception until May 2024 without any search restrictions. The detailed search approach and results are outlined in (Table S1).

### 2.3 Eligibility criteria

We included randomized clinical trials (RCTs), with the following PICOs criteria:

Population (P); patients with ischemic heart disease and previous ICD shocks; intervention (I); catheter ablation; comparator (C); antiarrhythmic drugs OR no catheter ablation; outcome (O); the primary outcomes of this review were the VT Storms and Ventricular tachycardia/Ventricular fibrillation (VT/VF) recurrence. The secondary outcomes included the other efficacy outcomes, including the incidence of appropriate ICD therapy, any ICD shocks, appropriate ICD shocks, inappropriate ICD shocks, cardiac hospitalization, and Heart failure (HF) hospitalization. The safety outcomes include any adverse events, any serious adverse events, all-cause mortality, and syncope.

### 2.4 Study selection

Search results from all the databases were imported to Covidence.org, and duplicates were removed automatically. The remaining records were screened independently by four authors (U.K., A.M.A., M.I., & J.A.), and any conflict between them was resolved by another author (C.R.B). The screening was done in two steps: (I) title and abstract screening to determine the relevance of the study for this meta-analysis, and (ii) full-text screening according to the inclusion criteria for the final eligibility for qualitative and quantitative analysis.

### 2.5 Data extraction

Data was collected independently by four review authors (M.A., M.H.K., J.B., & W.R.) and extracted into a uniform data extraction Excel sheet. The extracted data included characteristics of the included studies, including first author name, year of publication, country, study design, total participants, type of ablation, control technique and power used, inclusion criteria, primary outcome and follow-up duration; participants' baseline characteristics, including the number of participants, mean age, gender, body mass index (BMI), left ventricular ejection fraction (LVEF), New York Heart Association (NYHA) functional class, previous myocardial infarction (MI) and associated co-morbidities; and outcome measures as previously described across the intervention and comparator group. Any disagreement was resolved by consensus.

### 2.6 Risk of bias and certainty of evidence

The quality assessment of studies was independently conducted using the Cochrane RoB2 tool [13] by (M.A., M.S.R, J.B., & W.R). Moreover, they evaluated five domains, including deviation from the intended intervention, the risk of bias linked to the randomization process, outcomes measuring, missing outcome information, and choosing the reported outcomes and results. Any conflicts were resolved by consensus and discussion.

### 2.7 Statistical analysis

RevMan (version 5.3; Copenhagen: The Nordic Cochrane Centre, The Cochrane Collaboration, 2014) software was used for statistical analysis. The analysis combined results from multiple studies using either risk ratios (for dichotomous outcomes) or mean differences (for continuous outcomes), both with 95 % confidence intervals. A random-effects model was applied when significant heterogeneity (I^2^ > 50 %) was detected using the Chi-square and I-square tests; otherwise, a common-effect model was used. Heterogeneity was interpreted according to the Cochrane Handbook (chapter nine) [12], with an I^2^ value of 0–40 percent indicating low heterogeneity, 30–60 percent signifying moderate heterogeneity, 50–90 percent may represent substantial heterogeneity, and 75–100 percent signifying considerable heterogeneity. A Chi-square test p-value below 0.1 was considered statistically significant for heterogeneity.

## 3 Results

### 3.1 Search results and study selection

**Fig. 1:**
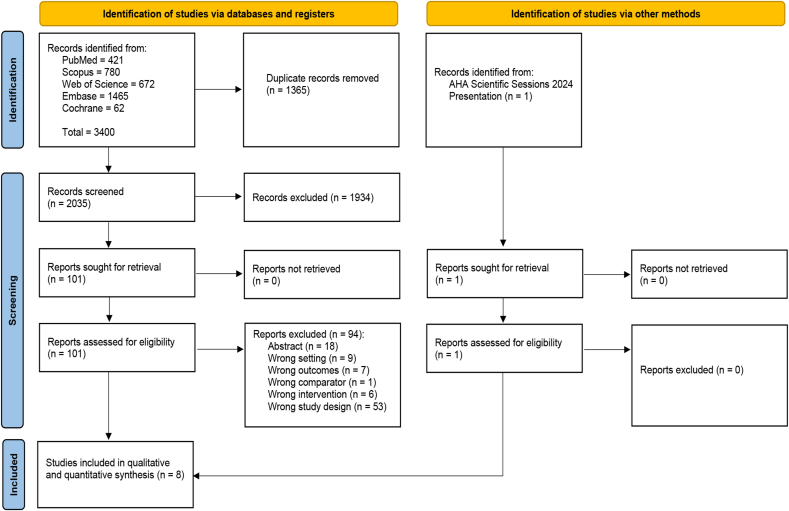
PRISMA flow chart of the screening process.

A total of 3400 records were incorporated from five databases into Covidence. Of these, 1365 were duplicates and were automatically removed, leaving 2035 records for screening. Out of these, 1934 records were deemed irrelevant and excluded during the title and abstract screening, resulting in 101 studies for full-text review. One RCT was identified through manual searching on PubMed. Eight RCTs met the inclusion criteria and were included in qualitative and quantitative synthesis (Fig. 1).

### 3.2 Characteristics of included studies

Eight RCTs [6,8,[14], [15], [16], [17], [18], [19]] involving 1252 Patients fulfilled the inclusion criteria, of which 619 were allocated to the ablation group and 633 to the control group. Table 1 summarize the main characteristics of the included RCTs.

**Table 1:** Summary of the characteristics of the included studies.

Study ID	Study Design	Country	NCT number	Total Participants	Intervention (Catheter ablation) Description	Control	Preventive or Abortive Ablation	Main Inclusion Criteria	Primary Outcome	Follow-up duration
Sapp et al., 2024 (VANISH 2) [19]	Open-label, multicenter, randomized controlled trial	International	NCT02830360	416	Catheter ablation procedures were conducted according to a standardized approach, which included the induction of ventricular tachycardia and electroanatomic mapping of the ventricular substrate potentially responsible for ventricular tachycardia, with delivery of radiofrequency energy at the potentially arrhythmic substrate to render ventricular tachycardia non-inducible.	Patients eligible for sotalol received 120 mg orally twice daily, while those ineligibles were treated with amiodarone. Amiodarone therapy started at 400 mg orally twice daily for 2 weeks, continued at 400 mg daily for 4 weeks, and was then maintained at 200 mg daily.	Abortive	Patients were eligible if they had a history of myocardial infarction and experienced at least one VT event in the past six months while not receiving antiarrhythmic treatment. Eligible events included sustained monomorphic VT requiring termination by ICD shock, medication, or cardioversion; three or more sustained VT episodes within 24 h; three or more VT episodes, including at least one symptomatic episode, treated with ICD anti-tachycardia pacing; or five or more episodes of monomorphic VT, regardless of symptoms.	Composite of death from any cause during follow-up or, more than 14 days after randomization, VT storm, appropriate ICD shock, or sustained VT treated by medical intervention	66 months
Al-khatib et al., 2014 (CALYPSO) [14]	Open-label, multicenter, randomized controlled trial	United States	NA	27	Catheter ablation within one month aimed for non-inducibility of VT, using the FDA-approved NaviStar ThermoCool catheter. Endocardial ablation was preferred, with epicardial allowed if needed. Mapping methods were physician-determined based on VT stability.	2006 ACC/AHA guidelines were followed, allowing amiodarone and sotalol as first-line therapies, with mexiletine, ranolazine, and dofetilide as second-line options. Flecainide, propafenone, procainamide, and disopyramide were not permitted.	Abortive	18 years old having ICD, with or without cardiac resynchronization therapy, Wall motion abnormalities, and coronary artery disease with ≥70 % stenosis in one major coronary artery.	Feasibility	6 months
Arenal et al., 2022 (SURVIVE-VT) [15]	Double-blinded, multicenter, randomized controlled trial	Spain	NCT03734562	144	The ablation procedures were performed using an endocardial substrate-based approach aimed at eliminating all the arrhythmogenic substrates, avoiding VT induction.	Unless contraindicated, patients in the AAD group were treated with amiodarone beta-blockers, those with contraindication to amiodarone were treated with sotalol beta-blockers, and those with contraindication or intolerance to beta-blockers received only amiodarone.	Abortive	Previous MI > six weeks ago. Medical Treatment: Optimal treatment if ventricular dysfunction is present. Symptomatic VT Episodes: Sustained VT treated with ICD shock within the last six months. Sustained VT with syncope, even if terminated with anti-tachycardia pacing.	Cardiovascular death, appropriate ICD shock, unplanned hospitalization for worsening heart failure, or severe treatment-related complications from enrollment up to the 24-month follow-up.	36 months
Kuck et al., 2010 (VTACH) [16]	Single-blinded, multicenter, randomized controlled trial	International	NCT00919373	107	The intervention group underwent mapping and ablation using either an electroanatomic system (Carto) or a non-contact system (Ensite), following standard mapping criteria for stable VT and lesion design for substrate modification in non-inducible or unstable VT.	All patients received cardioverter-defibrillators from St Jude Medical with specific programming parameters.	Abortive	Patients were aged 18–80 years with ICD indication for secondary prevention after stable clinical VT without reversible cause, having coronary artery disease and previous myocardial infarction. The LVEF was ≤50 %, measured by echocardiography or contrast ventriculography.	Time from defibrillator implantation to recurrence of any sustained VT or VF.	24 months
Kuck et al., 2017 (SMS) [17]	Open-label, multicenter, randomized controlled trial	International	NCT00170287	111	Catheter ablation was performed before ICD implantation, with attempts made to ablate all inducible VTs. Three mapping and ablation strategies were available: CARTO electroanatomic system mapping, EnSite NavX system mapping, and conventional mapping.	ICDs were implanted according to current standards, with specific recommendations for R-wave amplitude, pacing threshold, intraoperative induction of VF episodes terminated by shocks, and programming of VF and VT zones.	Abortive	Patients were aged 18–80 years having coronary artery disease and LVEF ≤40 %. Clinically unstable VT, cardiac arrest, or syncope with VT inducible at baseline.	Time to first recurrence of VT/VF.	33 months
Reddy et al., 2007 (SMASH-VT) [6]	Open-label, multicenter, randomized controlled trial	International	ISRCTN62488166.	128	During the catheter ablation for VT, femoral access was gained, and VT was induced via programmed stimulation, sometimes with antiarrhythmic drugs. Electro anatomical mapping using CARTO identified arrhythmogenic infarct regions by voltage amplitude. Pace mapping during sinus rhythm helped locate VT exit sites, where linear ablation lesions were made, prioritizing ≤1.0 mV border zones to minimize ventricular dysfunction. Post-ablation programmed stimulation was repeated to assess efficacy.	ICDs were implanted per guidelines, ensuring adequate R-wave amplitude and pacing threshold. Intraoperative VF induction was performed, followed by programming a VF zone (200–220 bpm, shock therapy only) and a VT zone (ATP and shock) detecting at least 16 beats. VT zone parameters varied based on rate: 160–180 bpm for rates >220 bpm or 60 ms above the slowest VT for rates <220 bpm.	Preventive	Patients were Adults (≥18) with MI history, documented by ECG/cardiac imaging, with recent/planned ICD implantation for: VF arrest, Syncope with inducible VT, Primary prophylaxis ICD with appropriate therapy.	Survival free from any appropriate ICD therapy.	24 months
Sapp et al., 2016 (VANISH) [8]	Open-label, multicenter, randomized controlled trial	International	NCT01456060	259	The procedure included venous and arterial access, advancement of electrode catheters to the right ventricle and His bundle positions, and VT induction with programmed ventricular stimulation from two ventricular sites at two drive cycle lengths with up to triple ventricular extra stimulation, coupled not closer than 180 msec. If clinical VT was not induced, isoproterenol could be administered 0.5–10 mcg/min in a dose adjusted to achieve a 30 % increase in baseline heart rate.	Patients in an escalated-therapy group experiencing arrhythmias despite taking less than 300 mg of amiodarone daily received a loading dose of 400 mg twice daily for two weeks, followed by 400 mg daily for one week, then 300 mg daily thereafter. For those on at least 300 mg of amiodarone, mexiletine (200 mg three times daily) was added. Patients initially on other antiarrhythmics were switched to 400 mg amiodarone twice daily for two weeks, followed by 400 mg per day for four weeks, and 200 mg per day afterward.	Abortive	Patients were eligible if they had a prior myocardial infarction, an implantable defibrillator, recent VT events, and had "failed" first-line antiarrhythmic therapy. Failure was defined as experiencing VT or ICD therapy despite adequate treatment with amiodarone (≥10 g) or other antiarrhythmics for at least 2 weeks.	Incidence of VT.	24 months
Žižek et al., 2024 (PREVENTIVE VT) [18]	Open-label, multicenter, randomized controlled trial	Europe	NCT03445096	60	Catheter ablation was performed before ICD implantation, utilizing high-density voltage mapping to delineate myocardial scars and border zones in the LV. Abnormal electrograms (EGMs) were identified and targeted for ablation using a contact force sensing catheter. The procedure aimed to eliminate these abnormal EGMs and achieve VT non-inducibility, verified through remapping and programmed stimulation. No time limits were set for mapping or ablation, allowing operator discretion throughout the process.	Single-chamber ICD devices were recommended unless indicated otherwise (e.g. dual-chamber ICD device or cardiac resynchronization ICD device). The physician had the discretion to select the manufacturer and type of the device. Uniform primary prevention device settings for arrhythmia detection and therapy from different manufacturers were recommended.	Preventive	Patients were eligible for inclusion if they had primary prevention indication for ICD with LVEF ≤40 % despite optimal medical therapy, had angiographically proven coronary chronic total occlusion that was associated with previous myocardial infarction.	Incidence of VT.	36 months

VT: ventricular tachycardia, LVEF: left ventricular ejection fraction, ICD: intracardiac cardioverter defibrillator.

mg: milligram, bpm: beats per minute.

### 3.3 Risk of bias

Among the eight studies reviewed, six were categorized as having a low risk of bias regarding the five domains [6,8,15,16,18]. Two studies Kuck et al., 2017 [17] and Al-Khatib et al., 2014 [14] were assessed to have some concerns in the randomization and allocation process.

**Fig. 2:**
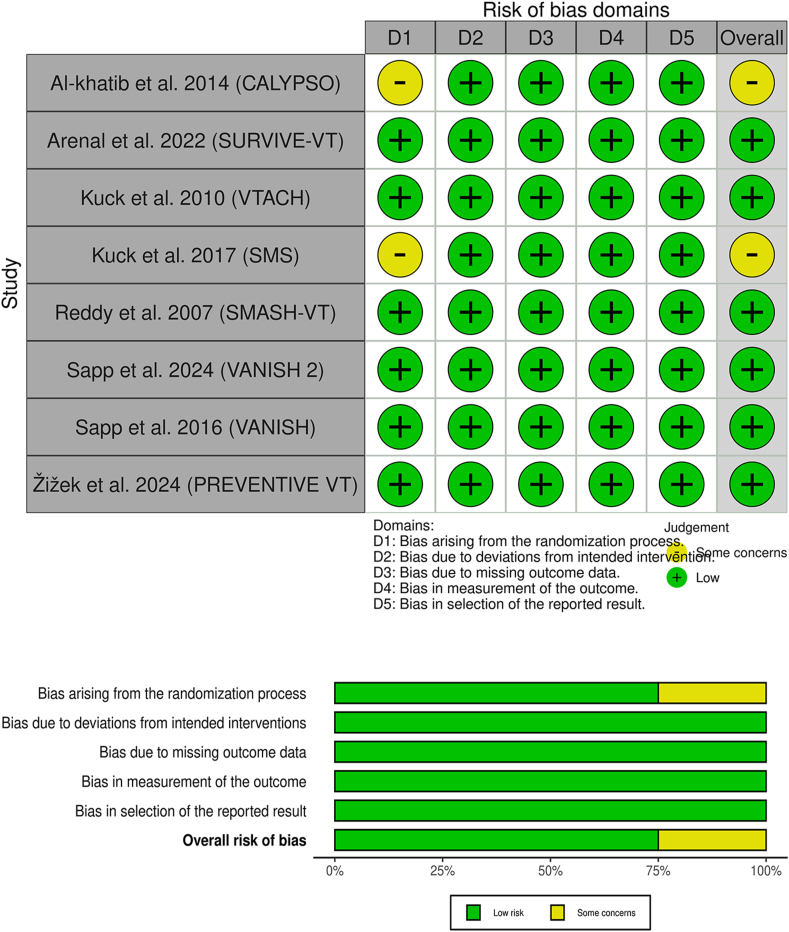
Quality assessment of risk of bias in the included trials. The upper panel presents a schematic representation of risks (low = green, unclear = yellow, and high = red) for specific types of biases of each of the studies in the review. The lower panel presents risks (low = green, unclear = yellow, and high = red) for the subtypes of biases of the combination of studies included in this review. (For interpretation of the references to colour in this figure legend, the reader is referred to the Web version of this article.)

The risk of bias for each of the included studies is shown in (Fig. 2).

### 3.4 Primary outcomes

#### 3.4.1 VT storms

**Fig. 3:**
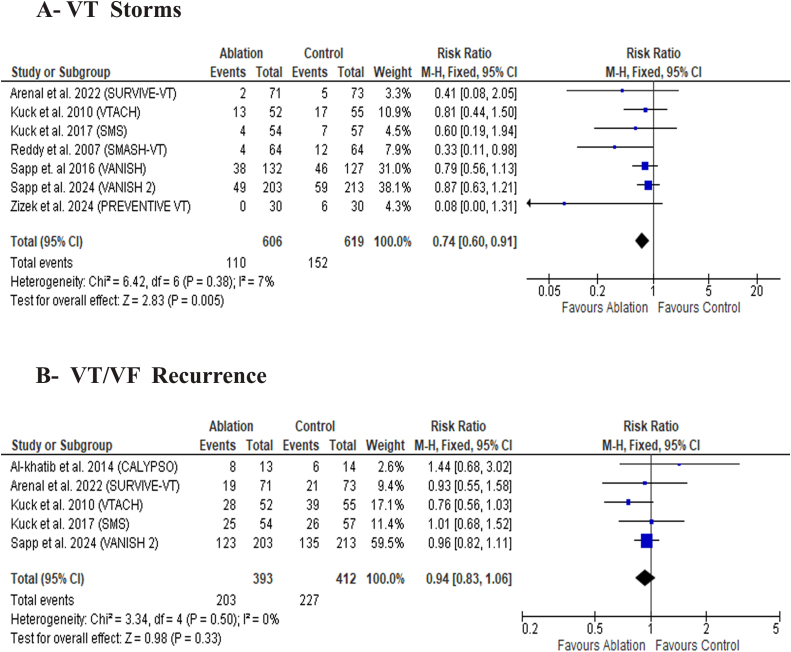
Forest plot of the primary efficacy outcomes, MD: mean difference, RR: risk ratio, CI: confidence interval.

Catheter ablation was significantly associated with a lower risk of VT storms compared to the control group **[**RR: 0.74 with 95 % CI (0.60, 0.91), P = 0.005) (Fig. 3-A). Pooled studies were homogeneous for VT storms (I^2^ = 7 %, P = 0.38).

#### 3.4.2 VT/VF recurrence

Catheter ablation did not show any significant difference over the control group in VT/VF recurrence [RR: 0.94 with 95 % CI (0.83, 1.06), P = 0.33) (Fig. 3-B).

Pooled studies were homogeneous for VT/VF recurrence (I^2^ = 0 %, P = 0.50).

### 3.5 Secondary outcomes

#### 3.5.1 ICD therapy and ICD shocks

**Fig. 4:**
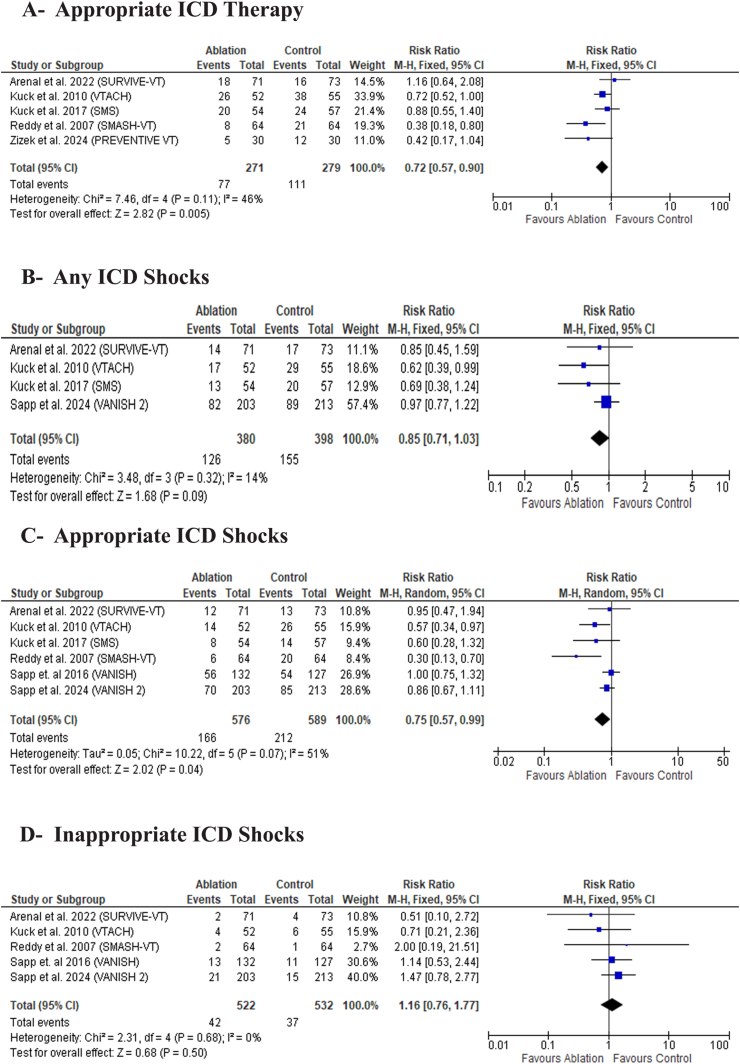
Forest plot of the secondary efficacy outcomes, MD: mean difference, RR: risk ratio, CI: confidence interval.

Catheter ablation was significantly associated with a lower incidence of appropriate ICD therapy [RR: 0.72 with 95 % CI (0.57, 0.90), P = 0.005) (Fig. 4-A), while there was no significant difference in any ICD shocks [RR: 0.85 with 95 % CI (0.71, 1.03), P = 0.09) (Fig. 4-B). Catheter ablation was significantly associated with a lower incidence of appropriate ICD shocks [RR: 0.75 with 95 % CI (0.57, 0.99), P = 0.04) (Fig. 4-C); however, there was no significant difference in inappropriate ICD shocks between the two groups [RR: 1.16 with 95 % CI (0.76, 1.77), P = 0.50) (Fig. 4-D).

Pooled studies were homogeneous in appropriate ICD therapy (I^2^ = 46 %, P = 0.11), any ICD shocks (I^2^ = 14 %, P = 0.32), and inappropriate ICD shocks (I^2^ = 0 %, P = 0.68). However, Pooled studies were heterogeneous in appropriate ICD shocks (I^2^ = 51 %, P = 0.07). Sensitivity analysis revealed that the heterogeneity was best resolved after excluding studies by Sapp et al., 2016 (I^2^ = 47 %) (Fig. S1) and Reddy et al., 2007 (I^2^ = 7 %) (Fig. S2).

#### 3.5.2 Hospitalization

**Fig. 5:**
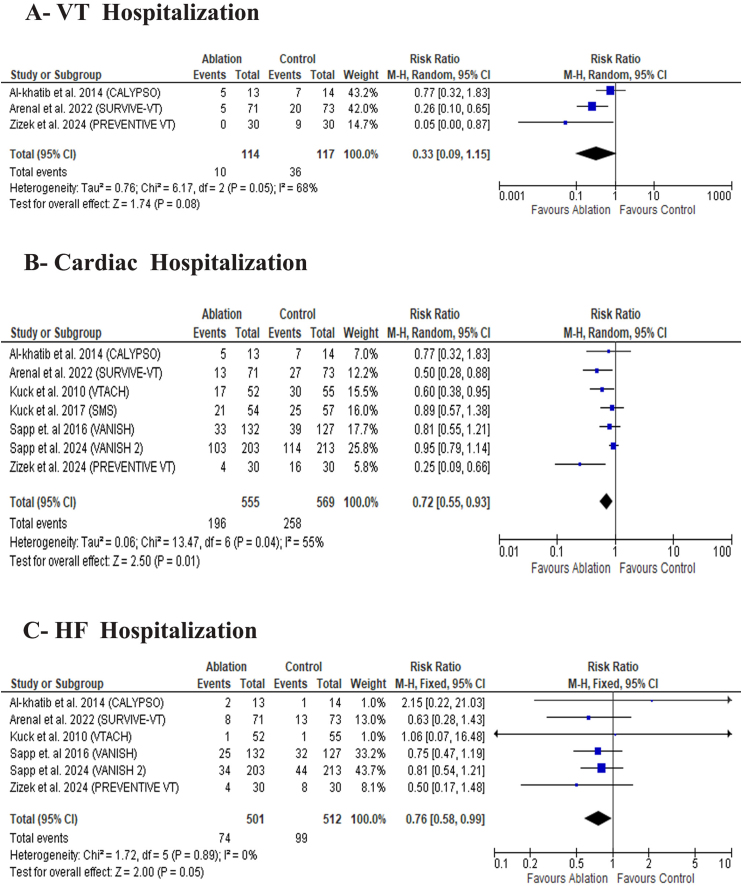
Forest plot of the secondary efficacy outcomes, MD: mean difference, RR: risk ratio, CI: confidence interval.

There was no difference between the two groups in VT hospitalization [RR: 0.33 with 95 % CI (0.09, 1.15), P = 0.08) (Fig. 5-A). However, catheter ablation was significantly associated with a lower incidence of cardiac hospitalization [RR: 0.72 with 95 % CI (0.55, 0.93), P = 0.01) (Fig. 5B) and lower incidence of HF hospitalization [RR: 0.76 with 95 % CI (0.58, 0.99), P = 0.05) (Fig. 5C).

The pooled studies were homogeneous in HF hospitalization (I^2^ = 0 %, P = 0.89). However, the studies were heterogeneous in VT hospitalization (I^2^ = 68 %, P = 0.05) and cardiac hospitalization (I^2^ = 55 %, P = 0.04).

The heterogeneity in VT hospitalization was best resolved by excluding Al-khatib et al., 2014 (I^2^ = 18 %) (Fig. S3). Heterogeneity in cardiac hospitalization was best resolved by excluding Sapp et al., 2024 (I^2^ = 37 %) (Fig. S4) and Zizek et al., 2024 (I^2^ = 31 %) (Fig. S5).

#### 3.5.3 Safety outcomes

**Fig. 6:**
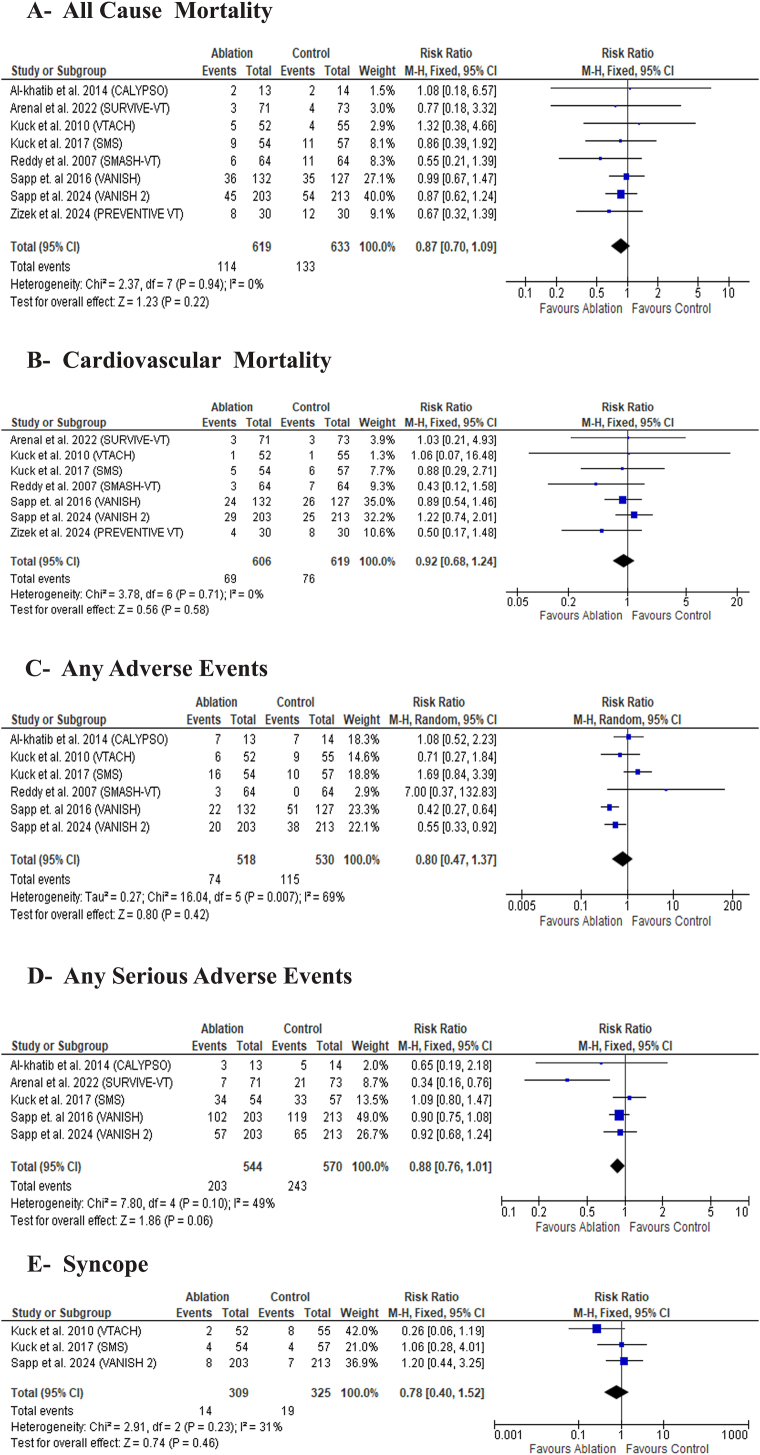
Forest plot of the safety outcomes, RR: risk ratio, CI: confidence interval.

Catheter ablation did not show any significant difference over medical therapy in all-cause mortality [RR: 0.87 with 95 % CI (0.70, 1.09), P = 0.22) (Fig. 6A), cardiovascular mortality [RR: 0.92 with 95 % CI (0.68, 1.24), P = 0.58) (Fig. 6B). Additionally, there was no significant difference between the two groups in any adverse events [RR: 0.80 with 95 % CI (0.47, 1.37), P = 0.42) (Fig. 6C) any serious adverse events [RR: 0.88 with 95 % CI (0.76, 1.01), P = 0.06) (Fig. 6D), and syncope [RR: 0.78 with 95 % CI (0.40, 1.52), P = 0.46) (Fig. 6E).

The pooled studies were homogeneous in all-cause mortality (I^2^ = 0%, P = 0.94), cardiovascular mortality (I^2^ = 0%, P = 0.71),6 any serious adverse events (I^2^ = 49 %, P = 0.10), and syncope (I^2^ = 31 %, P = 0.23). Significant heterogeneity was observed in any adverse events across the included studies (I^2^ = 69%, P = 0.007), which persisted despite the exclusion of individual studies.

## 4 Discussion

### 4.1 Summary of the findings

This is a systematic review and meta-analysis of eight RCTs that aimed to assess the impact of ablation therapy on the outcomes of IHD-related VT in patients receiving ICD implantation. The compared cohorts underwent either an ablation procedure with ICD or an ablation free modality that included ICD with or without AADs (standard care). We found with a moderate level of certainty that ablation has some important advantages over standard therapy, including a reduction in (i) the risk of VT storms but not VT/VF recurrence, (ii) the incidence of appropriate ICD therapy and appropriate ICD shocks but not inappropriate or any ICD shocks, and (iii) the incidence of cardiac and HF hospitalization but not VT hospitalization. Additionally, regarding safety, patients undergoing ablation showed no difference in susceptibility to all-cause mortality, cardiovascular mortality, serious or any adverse events, and syncope compared to those with an ablation-free strategy.

Our findings agree with the previous Martinez et al. meta-analysis in which the authors compared the outcomes of patients with IHD-related VT treated with or without catheter ablation [20]. Using records of five RCTs, they demonstrated lower odds of appropriate ICD therapies, appropriate ICD shocks, VT storms, and cardiac hospitalizations among ablation groups with respect to their counterparts. However, this study was conducted in 2019, and since then, few RCTs have been published, including SURVIVE-VT in 2022 [15], PREVENTIVE-VT in 2024 [18], and VANISH 2 in 2024 [19]. Therefore, an updated analysis was needed.

### 4.2 Mechanism of VT in IHD

Following myocardial infarction, an arrhythmogenic substratum can be created within the surviving myocardial strands trailing or surrounding the post-infarct ventricular scar [21,22]. This produces a reentrant arrhythmia, where local electrical conduction occurs in circuits or loops [23]. Different electrophysiological alterations are found diffusely in the arrhythmogenic scar area, a heterogeneous tissue of completely unexcitable fibrosis and a thin layer of surviving myocytes. These involve impairment of the conduction velocity due to rarefaction of gap junctions and defects in myocyte excitability. Moreover, enhanced sympathetic tone results in decreased action potential duration, increased sarcoplasmic reticulum calcium leak, and triggered activity, causing premature stimulation [21,23].

### 4.3 Antiarrhythmic drugs are less effective than ablation for the prevention of IHD-related VT

Four RCTs compared treatment with ablation versus escalating AADs as adjuvant modalities to ICD insertion. All four showed lower efficacy of AADs [8,15,19,24]. Amiodarone, often in combination with β-blockers (e.g., sotalol), is the cornerstone of antiarrhythmic therapy for VT secondary to IHD [25]. While ablation aims to destroy the excitable arrhythmogenic myocardium, often by radiofrequency energy irreversibly [26], the goals of antiarrhythmics are only to reduce the incidence and/or the gravity of acute arrhythmia episodes [27].

During ischemic cardiomyopathy, the nature of arrhythmia per se may play a key role in the observed inferiority of antiarrhythmic medication. Thus, increased amounts of cardiac fibrosis (the main pathogenic element in IHD-related VT) were previously shown to alter the antiarrhythmic effects of amiodarone and sotalol [28]. Moreover, one study revealed that long-term treatment with amiodarone reduces slow, delayed rectifier K+ current (IKs), while short-term treatment promotes it [29]. IKs have a protective role against ventricular arrhythmias through their resistance to excessive prolongation of ventricular action potential and prevention of early afterdepolarizations in ventricular myocytes [30]. Therefore, its inhibition would favor ventricular arrhythmogenesis. In line with this, the short-term administration of amiodarone leads to rapid delayed rectifier K+ current (IKr) channel inhibition, an additional mechanism by which amiodarone can prevent VT/torsade de pointes [31]. Nevertheless, this action appears lost in long-term use [29]. This suggests that amiodarone may lose some of its anti-VT action over time, thus leading to treatment failure. Such a phenomenon may be explained by the accumulation of amiodarone and its major active metabolite, desmethyl-amiodarone (DEA), in plasma and tissue, which contributes to the modulation of the drug's chronic effects or even their paradoxicality [32]. Knowing that amiodarone is metabolized by the liver [33] and that IHD patients have an increased incidence of hepatic disease [34], these patients may be more susceptible to amiodarone accumulation and subsequent decrease in its pharmacological action against VT. Since amiodarone was found to increase the defibrillation thresholds, especially during ischemic events, significantly. Subsequently, recommendations were released on the necessity of reassessing the defibrillation thresholds after initiation of antiarrhythmic drug therapy [35].

Our analysis revealed some surprising findings. For instance, although catheter ablation use was associated with reduced appropriate ICD therapies, it did not affect the incidence of VT recurrences. This could be due to the difference in VT severity and sustainability during recurrences. Thus, the two groups may have developed recurrent VT with similar incidence but lesser severity in the catheter ablation group, notably requiring less use of necessary (appropriate) ICDs.

Furthermore, despite lowering the VT storm risk, catheter ablation did not affect the inappropriate or any ICDs outcomes. Inappropriate ICDs use is reflective of the total number of inappropriate, unnecessary shocks delivered regardless of the number of VT events [15]. This outcome is dependent on factors other than VT frequency or severity, such as age, history of atrial fibrillation [36], and possibly the physician's approach for ICDs shocks delivery during arrhythmias occurrence. In the context of clinical trials, such an approach could be more rational and rigorous than in actual practice regardless of the adjuvant anti arrhythmic treatment strategy. Therefore, the superior effect of catheter ablation may have manifested only when VT was severe enough to indicate ICDs shock which corresponds to appropriate ICDs.

Cardiac hospitalization was referred to as hospitalization due to any cardiac reason, including heart failure. Since heart failure hospitalizations were less in the catheter ablation group (perhaps due to a better LV function at baseline as was the case for the SURVIVE VT trial [15] it could explain why they had an overall lower incidence of cardiac hospitalizations.

Antiarrhythmic drugs also expose the risks of side effects in IHD patients. For instance, amiodarone can cause a variety of pulmonary, thyroid, hepatic, cardiac, neurological, and skin adverse events, many of which can be serious (e.g., pulmonary fibrosis, hepatitis, QTc prolongation, and others) [37]. Sotalol can aggravate existing arrhythmias or induce new arrhythmias. [38]. Previously, it was shown that ablation procedures appear to display a good safety profile overall [26]. This was supported by our study, as there were comparable risks of safety outcomes between ablation-based and ablation-free modalities, especially in terms of all-cause and cardiovascular mortality. These findings indicate the real benefit of catheter ablation intervention without exposing the patient to notable safety concerns.

### 4.4 Clinical implications

In patients with IHD, VT ablation is more effective than standard care in preventing severe arrhythmia episodes, such as VT storms and appropriate ICD shocks. Although ablation reduces these life-threatening events, it does not completely prevent all VT episodes from recurring. This suggests that although VT ablation is advantageous, it remains non-curative and thus not entirely satisfactory. Hence, there is a need for procedural (technical) and non-procedural strategies to enhance the efficacy of this intervention. For instance, considering the mechanism of VT during IHD, ablation therapy has the best chance of eliminating the arrhythmogenic substratum if it is performed on the isthmus of the reentry circuit (aka, the diastolic corridor or central common pathway) where all loops rejoin [23]. However, achieving electrophysiological assessment of complex VT substrates, including the isthmus, is often impossible in clinical practice due to the non-inducibility/intolerability of arrhythmias, which interrupts the mapping and compromises the therapeutic results [23,39]. This obstacle may be overcome by developing more sophisticated high-resolution mapping technologies like ripple mapping and novel electrogram algorithms [40,41]. Besides unmappable substrates, another reported reason that reduces VT eradication after ablation is procedural challenges such as epicardial or intramural free wall VT, deep septal VT, and VT proximal to sensible anatomic structures (the bundle of His, left phrenic nerve, or a coronary artery) ultimately favoring acute ablation failure and arrhythmia persistence [42]. These anatomic challenges may be more manageable with advanced ablation approaches such as epicardial ablation via a subxiphoid epicardial window, trans-coronary ethanol ablation, and surgical ablation [42].

Finally, ablation is not perfectly safe, as complications of endocardial mapping are rare but possible [26] (the mapping in VT associated with ischemic cardiomyopathy is typically endocardial because the scar region is mostly endocardial) [27]. These include damage to the aortic valve or a coronary artery ostium, vascular access complications, and cerebral or systemic embolism. Epicardial mapping exposes even more concerns, such as injury to the epicardial coronary arteries [26]. Careful preprocedural measures, technical ameliorations, and enhanced expertise can prevent such compilations.

### 4.5 Study limitations

A primary limitation of this meta-analysis was the insufficient number of the included RCTs (eight RCTs with a total of 1252 participants), significantly minimizing the robustness and generalizability of the findings. The follow-up period was, in most studies, about two years, which allows exclusive evaluation of the short and medium-term outcomes. This underscores the necessity for more extensive studies to evaluate the interventions' long-term effects. Another weakness was the presence of heterogeneities in some secondary endpoints, such as appropriate ICD shocks, VT hospitalization, cardiac hospitalization and any adverse events. This was likely due to differences in the patient's baseline characteristics (e.g., type of IHD), treatment protocols (ablation performed after or before ICD, antiarrhythmic medications added or not added to ICD, etc.), and follow-up duration.

## 5 Conclusion

The current evidence shows that, in patients with sustained VT secondary to IHD, the addition of ablation therapy to ICD appears to display a superior efficacy but a similar safety profile to standard ablation-free modality. Hence, by comparing the two strategies, adjuvant or neoadjuvant ablation was found to prevent better VT storms, appropriate ICD use, heart failure hospitalizations, and cardiac hospitalizations. On the other hand, no particular benefit of ablation-based approach over standard care was shown in terms of lowering VT/VF recurrences and VT hospitalizations. This suggests that adjuvant ablation therapy is potentially a cost-effective strategy to minimize the burden of IHD-related VT. Yet, improvements are still required to achieve better outcomes. Due to data scarcity, the above results can only be considered preliminary and need to be confirmed for more large-scale multicenter RCTs.

## Author contributions

U.K. conceived the idea. C.R.B., and J.B. designed the research workflow. J.A. and U.K. searched the databases. U.K., A.M.A., W.R., M.H.K., and M.I. screened the retrieved records, extracted relevant data, assessed the quality of evidence, and U.K. resolved the conflicts. A.A.I. performed the analysis. Y.K., Z.M., and M.S.R. wrote the final manuscript. K.C., and U.K. supervised the project. All authors have read and agreed to the final version of the manuscript.

## Ethics approval and consent to participate

Not applicable.

## Consent for publication

Not applicable.

## Availability of data and materials

The data is available upon reasonable request from the corresponding author.

## Funding

We received no funding for this study.

## Declaration of competing interest

The authors declare that they have no known competing financial interests or personal relationships that could have appeared to influence the work reported in this paper.
