# Supplementary material for: Catheter ablation versus medical therapy for ventricular tachycardia in patients with ischemic heart disease: A systematic review and meta-analysis of randomized controlled trials
Source: Indian Pacing Electrophysiol J. 2025 Mar 7;25(2):91–103. doi: 10.1016/j.ipej.2025.03.004 (PMC12138073; doi:10.1016/j.ipej.2025.03.004)
Supplement: Multimedia component 1 [file mmc1.docx]

**Catheter Ablation versus Medical Therapy for Ventricular Tachycardia in Patients with Ischemic Heart Disease: A Systematic Review and Meta-analysis of Randomized Controlled Trials.**

**Running title.**

Catheter Ablation for Ventricular Tachycardia.

**Authors.**

Ubaid Khan^1^, Yehya Khlidj^2^, Ahmed A. Ibrahim ^3^, Ahmed Mazen Amin^4^, Mohamed Saad Rakab^4^, Majd M. AlBarakat^5^, Muhammad Haris Khan^6^, Zuhair Majeed^7^, Muhammad Imran^8^, Junaid Ali^9^, Chet RanaBhat^10^, Wajeeh Ur Rehman^11^, Justin Brilliant^1^, Kashif Chaudhary^12^.

**Affiliations.**

1. Division of Cardiology, University of Maryland School of Medicine, Baltimore, MD, USA.
2. Faculty of Medicine, Algiers University, Alger Centre, Algeria.
3. Faculty of Medicine, Menoufia University, Menoufia, Egypt.
4. Faculty of Medicine, Mansoura University, Mansoura, Egypt.
5. Faculty of Medicine, Jordan University of Science and Technology, Irbid, Jordan.
6. Department of Medicine, Saidu Medical College, Swat, Pakistan.
7. Department of Medicine, King Edward Medical University, Lahore, Pakistan.
8. Faculty of Medicine, University College of Medicine and Dentistry, The University of Lahore, Lahore, Pakistan.
9. Department of Medicine, Saint Peter’s University Hospital, New Brunswick, NJ, USA.
10. Department of Medicine, University of Maryland Medical Center Midtown Campus, Baltimore, MD, USA,
11. Department of Internal Medicine, United Health Services, NY, USA.
12. Department of Electrophysiology, UPMC Williamsport, Pennsylvania, USA.

**Keywords.**

Ventricular Tachycardia, Ischemic Heart Disease, Catheter Ablation, Intracardiac Cardioverter Defibrillator, Meta-analysis.

**Corresponding author.**

Ubaid Khan,

Ubaidkhanafridi@yahoo.com

**Affiliation:**

Division of Cardiology, University of Maryland School of Medicine, Baltimore, MD, USA.

**Address:**

University of Maryland School of Medicine, Baltimore, MD, USA.

Phone # 001-443-813-6749.

**Legends**

**Tables.****Table S1:** Search strategy.

**Figures.**

**Figure S1:** Heterogeneity in Appropriate ICD shocks resolved after excluding Sapp et al. 2016.

**Figure S2:** Heterogeneity in Appropriate ICD shocks resolved after excluding Reddy et al. 2007.

**Figure S3:** Heterogeneity in VT- hospitalization resolved after excluding Al-khatib et al. 2014.

**Figure S4:** Heterogeneity in Cardiac hospitalization resolved after excluding Sapp et al. 2024.

**Figure S5:** Heterogeneity in Cardiac hospitalization resolved after excluding Zizek et al. 2024.

| Database | Search strategy | Search field | Search Results |
| --- | --- | --- | --- |
| PUBMED | (“Ventricular Tachycardia” OR “Idiopathic Ventricular Tachycardia” OR “Ventricular Tachyarrhythmia” OR “Nonsustained Ventricular Tachycardia” OR “Paroxysmal Supraventricular Tachycardia” OR “Supraventricular Tachycardia, Paroxysmal”) AND (“Cather ablation” OR “Ablation Catheter” OR “Transvenous Catheter Ablation” OR “Electrical Catheter Ablation” OR “Electric Catheter Ablation” OR “Transvenous Electric Ablation” OR “Catheter Ablation Radiofrequency” OR “Radiofrequency Catheter Ablation” OR “Percutaneous Catheter Ablation”) AND (“ICD” OR “Implantable Defibrillator” OR “Implantable Cardioverter Defibrillator” OR “Cardioverter Defibrillator Implantable” OR “Defibrillator Implantable Cardioverter” OR “Implantable Cardioverter”) AND (“ischemic heart” OR “ischemic cardiomyopathy” OR “ischemic heart disease” OR “ischemic disease”) | All fields | 421 |
| CENTRAL | (“Ventricular Tachycardia” OR “Idiopathic Ventricular Tachycardia” OR “Ventricular Tachyarrhythmia” OR “Nonsustained Ventricular Tachycardia” OR “Paroxysmal Supraventricular Tachycardia” OR “Supraventricular Tachycardia, Paroxysmal”) AND (“Cather ablation” OR “Ablation Catheter” OR “Transvenous Catheter Ablation” OR “Electrical Catheter Ablation” OR “Electric Catheter Ablation” OR “Transvenous Electric Ablation” OR “Catheter Ablation Radiofrequency” OR “Radiofrequency Catheter Ablation” OR “Percutaneous Catheter Ablation”) AND (“ICD” OR “Implantable Defibrillator” OR “Implantable Cardioverter Defibrillator” OR “Cardioverter Defibrillator Implantable” OR “Defibrillator Implantable Cardioverter” OR “Implantable Cardioverter”) AND (“ischemic heart” OR “ischemic cardiomyopathy” OR “ischemic heart disease” OR “ischemic disease”) | All fields | 62 |
| WoS | (“Ventricular Tachycardia” OR “Idiopathic Ventricular Tachycardia” OR “Ventricular Tachyarrhythmia” OR “Nonsustained Ventricular Tachycardia” OR “Paroxysmal Supraventricular Tachycardia” OR “Supraventricular Tachycardia, Paroxysmal”) AND (“Cather ablation” OR “Ablation Catheter” OR “Transvenous Catheter Ablation” OR “Electrical Catheter Ablation” OR “Electric Catheter Ablation” OR “Transvenous Electric Ablation” OR “Catheter Ablation Radiofrequency” OR “Radiofrequency Catheter Ablation” OR “Percutaneous Catheter Ablation”) AND (“ICD” OR “Implantable Defibrillator” OR “Implantable Cardioverter Defibrillator” OR “Cardioverter Defibrillator Implantable” OR “Defibrillator Implantable Cardioverter” OR “Implantable Cardioverter”) AND (“ischemic heart” OR “ischemic cardiomyopathy” OR “ischemic heart disease” OR “ischemic disease”) | All fields | 672 |
| Scopus | (“Ventricular Tachycardia” OR “Idiopathic Ventricular Tachycardia” OR “Ventricular Tachyarrhythmia” OR “Nonsustained Ventricular Tachycardia” OR “Paroxysmal Supraventricular Tachycardia” OR “Supraventricular Tachycardia, Paroxysmal”) AND (“Cather ablation” OR “Ablation Catheter” OR “Transvenous Catheter Ablation” OR “Electrical Catheter Ablation” OR “Electric Catheter Ablation” OR “Transvenous Electric Ablation” OR “Catheter Ablation Radiofrequency” OR “Radiofrequency Catheter Ablation” OR “Percutaneous Catheter Ablation”) AND (“ICD” OR “Implantable Defibrillator” OR “Implantable Cardioverter Defibrillator” OR “Cardioverter Defibrillator Implantable” OR “Defibrillator Implantable Cardioverter” OR “Implantable Cardioverter”) AND (“ischemic heart” OR “ischemic cardiomyopathy” OR “ischemic heart disease” OR “ischemic disease”) | All fields | 780 |
| EMBASE | (“Ventricular Tachycardia” OR “Idiopathic Ventricular Tachycardia” OR “Ventricular Tachyarrhythmia” OR “Nonsustained Ventricular Tachycardia” OR “Paroxysmal Supraventricular Tachycardia” OR “Supraventricular Tachycardia, Paroxysmal”) AND (“Cather ablation” OR “Ablation Catheter” OR “Transvenous Catheter Ablation” OR “Electrical Catheter Ablation” OR “Electric Catheter Ablation” OR “Transvenous Electric Ablation” OR “Catheter Ablation Radiofrequency” OR “Radiofrequency Catheter Ablation” OR “Percutaneous Catheter Ablation”) AND (“ICD” OR “Implantable Defibrillator” OR “Implantable Cardioverter Defibrillator” OR “Cardioverter Defibrillator Implantable” OR “Defibrillator Implantable Cardioverter” OR “Implantable Cardioverter”) AND (“ischemic heart” OR “ischemic cardiomyopathy” OR “ischemic heart disease” OR “ischemic disease”) | All fields | 1465 |

**Table 1: Search Strategy**


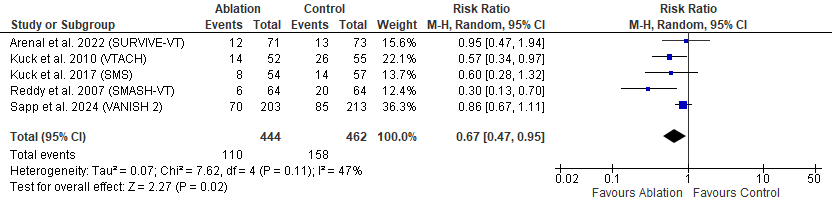


**Figure S1 shows heterogeneity in Appropriate ICD shocks resolved after excluding Sapp et al. 2016**

**
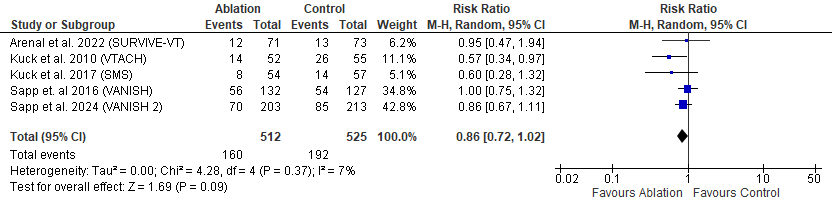
**

**Figure S2 shows heterogeneity in Appropriate ICD shocks resolved after excluding Reddy et al. 2007**


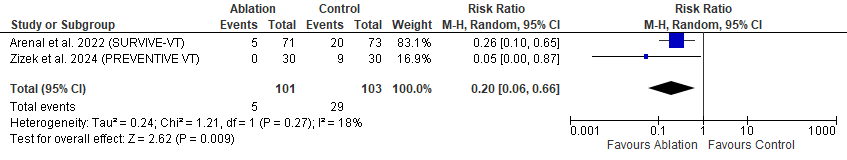


**Figure S3 shows heterogeneity in VT- hospitalization resolved after excluding Al-khatib et al. 2014**

**
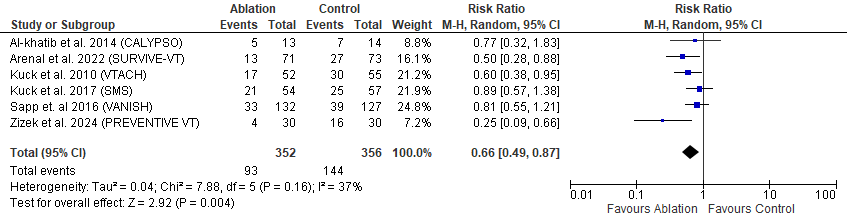
**

**Figure S4 shows heterogeneity in Cardiac hospitalization resolved after excluding Sapp et al. 2024**

**
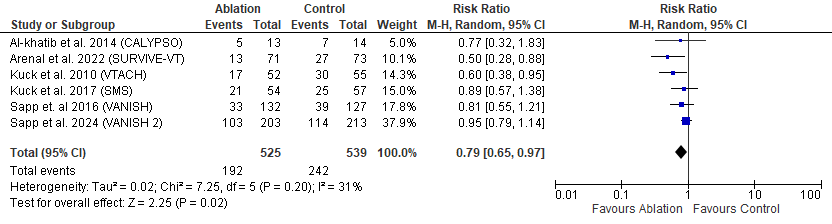
**

**Figure S5 shows heterogeneity in Cardiac hospitalization resolved after excluding Zizek et al. 2024**
