# Supplementary material for: Catheter ablation versus medical therapy for ventricular tachycardia in patients with ischemic heart disease: A systematic review and meta-analysis of randomized controlled trials
Source: Indian Pacing Electrophysiol J. 2025 Mar 7;25(2):91–103. doi: 10.1016/j.ipej.2025.03.004 (PMC12138073; doi:10.1016/j.ipej.2025.03.004)
Supplement: Multimedia component 2 [file mmc2.docx]

| **Study ID** | **Number of patients in each group** | | **Age (Years), Mean (SD)** | | **BMI, Mean (SD)** | | **Male, n (%)** | | **LVEF %, Mean (SD)** | | **NYHA functional class — no. (%)** | | | | | | **Previous MI, n (%)** | | **Comorbidities N. (%)** | | | | | | | | | | | | |
| --- | --- | --- | --- | --- | --- | --- | --- | --- | --- | --- | --- | --- | --- | --- | --- | --- | --- | --- | --- | --- | --- | --- | --- | --- | --- | --- | --- | --- | --- | --- | --- |
|  |  |  |  |  |  |  |  |  |  |  | **I** | | **II** | | **III** | |  |  | **Atrial fibrillation/flutter** | | **Hypertension** | | **Diabetes** | | **Previous PCI** | | **Previous CABG** | | **Stroke** | |  |
|  | **Intervention** | **Control** | **Intervention** | **Control** | **Intervention** | **Control** | **Intervention** | **Control** | **Intervention** | **Control** | **Intervention** | **Control** | **Intervention** | **Control** | **Intervention** | **Control** | **Intervention** | **Control** | **Intervention** | **Control** | **Intervention** | **Control** | **Intervention** | **Control** | **Intervention** | **Control** | **Intervention** | **Control** | **Intervention** | **Control** |  |
| **Sapp et al. 2024 (VANISH 2) [19]** | 203 | 213 | 67.7 (8.6) | 68.4 (8.0) | NA | NA | 193 (95.1) | 197 (92.5) | 34.0 (11.0) | 34.3 (10.3) | 87 (42.9) | 89 (41.8) | 99 (48.8) | 107 (50.2) | 17 (8.4) | 17 (8.0) | 203 (100) | 213 (100) | 70 (34.5) | 72 (33.8) | 160 (78.8) | 169 (79.3) | 79 (38.9) | 83 (39.0) | 128 (63.1) | 121 (56.8) | 82 (40.4) | 88 (41.3) | NA | NA |  |
| **Al-khatib et al. 2014 (CALYPSO) [14]** | 13 | 14 | 63 (30.7) | 63.7 (32.9) | NA | NA | 13 (100) | 12 (86) | 35 (41.5) | 26 (28.8) | 2 (22) | 3 (21) | 3 (33) | 5 (36) | 1 (11) | 3 (21) | NA | NA | 5 (39) | 4 (29) | 9 (69) | 12 (86) | 6 (46) | 5 (36) | 6 (46) | 8 (57) | 8 (62) | 8 (57) | NA | NA |  |
| **Arenal et al. 2022 (SURVIVE-VT) [15]** | 71 | 73 | 69.3 (9.1) | 70.3 (9.1) | 28 (4.8) | 27.8 (3.1) | 70 (98.6) | 68 (93.2) | 34 (11.4) | 32.7 (11.3) | 31 (44.3) | 31 (42.5) | 33 (47.1) | 37 (50.7) | 6 (8.6) | 5 (6.8) | 71 (100) | 73 (100) | 9 (13.6) | 8 (12.3) | 56 (78.9) | 47 (64.4) | 21 (29.6) | 15 (20.5) | 26 (38.2) | 26 (37.1) | 18 (26.5) | 12 (17.1) | NA | NA |  |
| **Kuck et al. 2010 (VTACH) [16]** | 52 | 55 | 67.7 (8.3) | 64.4 (8.2) | N/A | N/A | 50 (96) | 50 (91) | 34 (9.6) | 34.1 (8.8) | NA | NA | NA | NA | NA | NA | NA | NA | NA | N/A | NA | NA | NA | NA | 26 (50) | 24 (44) | 26 (50) | 22 (40) | NA | NA |  |
| **Kuck et al. 2017 (SMS) [17]** | 54 | 57 | 68.4 (7.7) | 65.9 (8.4) | NA | NA | 47 (87) | 46 (81) | 32.0 (6.9) | 30.4 (7.3) | NA | NA | NA | NA | NA | NA | 49 (96) | 56 (98) | NA | NA | NA | NA | NA | NA | 23 (46) | 25 (46) | 21 (41) | 24 (43) | NA | NA |  |
| **Reddy et al. 2007 (SMASH-VT) [6]** | 64 | 64 | 67 (9) | 66 (10) | NA | NA | 59 (92) | 52 (81) | 30.7 (9.5) | 32.9 (8.5) | NA | NA | NA | NA | NA | NA | NA | NA | NA | NA | 47 (73) | 43 (67) | 24 (38) | 32 (50) | NA | NA | NA | NA | 3 (5) | 8 (12) |  |
| **Sapp et al. 2016 (VANISH) [8]** | 132 | 127 | 67 (8.6) | 70.3 (7.3) | NA | NA | 123 (93.2) | 118 (92.9) | 31.1 (10.4) | 31.2 (10.7) | 33 (25.0) | 28 (22.0) | 69 (52.3) | 68 (53.5) | 30 (22.7) | 31 (24.4) | 132 (100) | 127 (100) | 52 (39.4) | 47 (37.0) | 92 (69.7) | 88 (69.3) | 37 (28.0) | 40 (31.5) | 50 (37.9) | 62 (48.8) | 63 (47.7) | 55 (43.3) | NA | NA |  |
| **Žižek et al. 2024 (PREVENTIVE-VT) [18]** | 30 | 30 | 65 (16) | 71 (10) | NA | NA | 29 (96.7) | 26 (86.7) | NA | NA | 1 (3.3) | 1 (3.3) | 20 (66.7) | 17 (56.7) | 9 (30) | 12 (40) | 13 (43.3) | 16 (53.3) | 9 (30) | 10 (33) | 24 (80) | 25 (83.3) | 8 (26.7) | 9 (30 | 13 (43.3) | 13 (43.3) | 1 (3.3) | 4 (13.3) | NA | NA |  |

**Table 2. Baseline characteristics of the population of the included study.**

*BMI: body mass index, SD: standard deviation, LVEF: Left ventricular ejection fraction,*

*NYHA: New york heart association functional classification, MI: Myocardial infarction*

*PCI: Percutaneous coronary intervention, CABG: Coronary artery bypass grafting*
